# Prevalence and Associations of Medical Expenditure Panel Survey–Defined Long COVID Among Adults: Cross-Sectional Study

**DOI:** 10.2196/92323

**Published:** 2026-06-22

**Authors:** David Rhys Axon, Stephanie Fenwick

**Affiliations:** 1 Division of Pharmacy Practice and Administrative Sciences, James L. Winkle College of Pharmacy University of Cincinnati Cincinnati, OH United States

**Keywords:** long COVID, social determinants of health, postacute sequelae of SARS-CoV-2, Andersen behavioral model, prevalence

## Abstract

**Background:**

Long COVID is a clinical condition that significantly influences quality of life, productivity, and morbidity in the individuals affected. Much of the research to date has examined medical comorbidities and their associations with long COVID, but there remains a substantial need to understand the social and behavioral factors associated with long COVID.

**Objective:**

The objective of this study was to investigate the prevalence and associations of Medical Expenditure Panel Survey (MEPS)–defined long COVID among adults in the United States through the application of the Andersen behavioral model.

**Methods:**

This cross-sectional database study used the 2022 MEPS dataset. Variables in this analysis were organized according to the Andersen behavioral model. The appropriate weighting variable was used to obtain weighted population-based estimates. Between-group differences (ie, those with MEPS-defined long COVID vs those without) were assessed using chi-square tests, and a multivariable binomial logistic regression model was developed to assess the association between each variable and having MEPS-defined long COVID.

**Results:**

A total of 11,266 individuals were eligible for inclusion in this study. This represented a weighted population of 256,500,584 American adults. Of these 11,266 individuals, 790 (7%; weighted population=18,397,214) had MEPS-defined long COVID, whereas 10,476 (93%; weighted population=238,103,371) did not. Variables identified that were statistically associated with having MEPS-defined long COVID among American adults included 3 predisposing variables (age, sex, and Asian race), 2 enabling variables (marital status and employment status), 3 need variables (number of chronic conditions, health status, and instrumental activity of daily living limitations), 1 personal health practices variable (ever receiving the COVID-19 vaccine), and 1 external environmental variable (south region).

**Conclusions:**

The prevalence and factors associated with having MEPS-defined long COVID among American adults in this study offer insights to expand our limited understanding of the complex environmental and social factors associated with MEPS-defined long COVID. Further research is required among the long COVID population to better understand and differentiate the causes and consequences of this condition.

## Introduction

Long COVID, or postacute sequelae of SARS-CoV-2, represents a clinical condition affecting certain individuals after an acute infection with SARS-CoV-2. Formal definitions of long COVID vary, and overall understanding of this disease state continues to evolve [1]. Broadly, long COVID is generally defined as a cluster of symptoms lasting a minimum of 1 to 3 months after acute SARS-CoV-2 infection (or probable infection) that cannot be otherwise attributed to another diagnosis [1]. Symptoms often present in a relapsing-remitting pattern and may include shortness of breath, fatigue, myalgias, and cognitive dysfunction. However, symptoms can vary widely between individuals, and multiple organ systems can be affected [1,2].

Long COVID places a substantial burden on the health care system and society as a whole. As of September 2024, the National Center for Health Statistics estimated that 17.9% of adults in the United States had experienced long COVID and 5.3% were currently experiencing it [3]. A systematic review and meta-analysis by Luo et al [4] examining the symptoms of long COVID determined that the mean duration of symptoms experienced by individuals with long COVID varied according to the symptom. Notably, shortness of breath lasted an average of 6 months; mental health symptoms lasted between 3 and 4 months on average; neurological symptoms such as fatigue, cognitive dysfunction, and memory difficulties lasted approximately 6 months on average; whereas word-finding difficulties and barriers to activity lasted an average of 12 months [4]. These prolonged symptoms can lead to meaningful productivity losses from the individuals affected. A 2025 study by Bartsch et al [5] used a computational simulation model to estimate the economic cost of long COVID. The researchers estimated that current long COVID cases would result in an overall cost to society of between US $2 billion and US $6.5 billion, with over 90% of these costs related to productivity losses. Moreover, the authors also reported an estimated loss of 35,000 to 121,000 quality-adjusted life years due to long COVID [5].

As long COVID is a relatively newly recognized disease process, insights into the pathophysiology, risk factors, prevention, and treatment are still evolving [1,2]. Understanding factors associated with long COVID is one area where more research is needed. Using data collected from 2020 to 2023 in the United States, Oelsner et al [6] investigated factors associated with persistent symptoms at 90 days after initial SARS-CoV-2 infection. Women and individuals with preexisting cardiovascular disease were more likely than other groups to have persistent symptoms 90 days after infection, whereas previous COVID-19 vaccination or infection with the Omicron SARS-CoV-2 variant were associated with lower likelihood of symptoms at 90 days [6]. In a large meta-analysis of international data by Hou et al [7], no previous COVID-19 vaccination, infection with an earlier SARS-CoV-2 variant (before Omicron), and female sex were identified as the most significant risk factors for long COVID. Other risk factors reported included presence of at least one comorbidity, cardiovascular disease, prior intensive care unit admission, hypertension, and chronic obstructive pulmonary disease [7]. Similarly, a 2023 meta-analysis of international study data specifically focusing on risk factors for long COVID also identified female sex and other medical comorbidities as risk factors. High BMI, smoking, and older age also increased the risk of long COVID, whereas vaccination was again protective [8].

Much of the current evidence on long COVID focuses on medical comorbidities as risk factors; however, there is a significant need for a more comprehensive understanding of factors associated with long COVID that include social determinants of health, environmental factors, and health behaviors [9]. An early analysis of factors associated with long COVID using data from the United Kingdom by Subramanian et al [10] identified multiple factors, such as belonging to an ethnic minority group, obesity, smoking, and socioeconomic deprivation, as being associated with an increased risk of long COVID. Similarly, a prospective observational study that focused on social determinants of health in people who developed acute COVID-19 in the United States found that factors such as food insecurity, financial hardship, experiences of medical discrimination, lower educational attainment, and living in a zip code with high household crowding were associated with an increased likelihood of developing long COVID compared with individuals without these factors [11]. Another 2025 analysis of US Household Pulse Survey data by Kim [12] noted that, compared with other groups, there was a higher risk of long COVID among Hispanic individuals; gay, lesbian, or bisexual individuals; and people with a low household income. Finally, a survey of people with a previous COVID-19 diagnosis in Germany by Kananian et al [13] reported that greater social connectedness was associated with less severe symptoms of long COVID. Although informative, these studies only examine a fraction of the social and behavioral factors potentially associated with long COVID. With so many social and behavioral characteristics unexplored, further research is warranted to develop a clearer concept of factors associated with long COVID.

The objective of this study was to investigate the prevalence and associations of long COVID among American adults through the application of the Andersen behavioral model (ABM) [14].

## Methods

### Study Design and Data

This cross-sectional database study used the 2022 Medical Expenditure Panel Survey (MEPS) dataset. The MEPS involves a series of panel-style interviews over a 2-year time frame using a subsample of individuals from the previous year’s National Health Interview Survey. The MEPS includes 3 core components: the household component, the medical provider component, and the insurance component. MEPS staff can use data from the medical provider component or the insurance component to supplement data in the household component. The 2022 full-year consolidated data file used in this study included data for 22,431 individuals and contained a wide variety of data related to personal demographic characteristics and health status. The MEPS is approved annually by an institutional review board, and all individuals provide informed consent before participating [15-17].

### Study Eligibility

Eligible individuals for this study included American adults (≥18 years of age) who were alive for the duration of the 2022 calendar year. Data for individuals under 18 years of age were not included in the MEPS data file to protect their anonymity. Individuals had to be alive for the full calendar year to ensure that they were within the scope for this analysis. Eligibility was determined using the person disposition status (MEPS variable: “PSTATS31/42/53”) and age (MEPS variable: “AGE22X”).

### Dependent Variable

The dependent variable in this study was having MEPS-defined long COVID. As part of the priority conditions enumeration section of the MEPS, participants are asked if they have ever been diagnosed with COVID-19 (MEPS variable: “COVIDEVER53”). If an individual has ever been diagnosed with COVID-19, subsequent questions are asked to determine whether the individual had symptoms lasting 3 months or longer that they did not have prior to having COVID-19 (MEPS variable: “LCEVER53”). If this was the case, then the individual was deemed by the MEPS to have long COVID. This is described as “MEPS-defined long COVID” for the purposes of this study. Although some variables related to COVID-19 have been included in the MEPS datasets since 2020, the 2022 dataset is the first time that a variable related to long COVID has been included. Therefore, this represents the first opportunity to investigate MEPS-defined long COVID in the nationally representative MEPS dataset [16,17].

### ABM Definition

#### Overview

**Figure 1:**
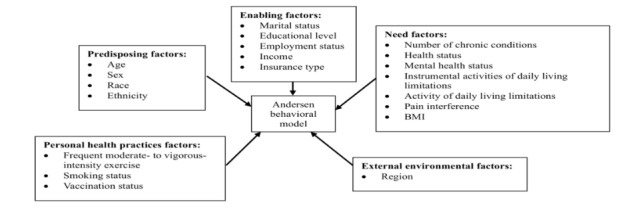
Variable framework.

Variables included in this analysis were organized according to the ABM [14]. Developed by Ronald M Andersen in 1968 and refined ever since, the ABM has been used for a variety of purposes in health service research, including as a way to organize variables associated with a particular outcome. The ABM as applied to this study consists of 5 categories: predisposing variables, enabling variables, need variables, personal health practices variables, and external environmental variables [14]. The MEPS data variable names are provided in parentheses after each variable in the text below [17] (Figure 1).

#### Predisposing Variables

In this study, predisposing variables included demographic characteristics that may be associated with MEPS-defined long COVID, such as age (≥65 years or 18-64 years; MEPS variable: “AGE22X”), sex (male or female; MEPS variable: “SEX”), race (White, Black, Asian, or other or multiple races; MEPS variable: “RACEV1X”), and ethnicity (Hispanic or not Hispanic; MEPS variable: “HISPANX”) [17].

#### Enabling Variables

In this study, enabling variables included nonbiological characteristics that facilitated engagement with health services to determine a diagnosis of COVID-19 and, subsequently, MEPS-defined long COVID, such as marital status (married; widowed, divorced, or separated; or never married; MEPS variable: “MARRY22X”), educational level (lower than high school, high school, or more than high school; MEPS variable: “EDUCYR”), employment status (employed or unemployed; MEPS variable: “EMPST53”), income (poor or low, middle, or high; MEPS variable: “POVCAT22”), and insurance type (any private, public only, or uninsured; MEPS variable: “INSCOV22”) [17].

#### Need Variables

In this study, need variables included characteristics that represented perceived and actual illness severity. This included the number of chronic conditions (0, 1, 2, 3, 4, or ≥5) from the following list: hypertension (MEPS variable: “BPMLDX”), coronary heart disease (MEPS variable: “CHDDX”), angina (MEPS variable: “ANGIDX”), myocardial infarction (MEPS variable: “MIDX”), other heart disease (MEPS variable: “OHRTDX”), stroke (MEPS variable: “STRKDX”), emphysema (MEPS variable: “EMPHDX”), chronic bronchitis (MEPS variable: “CHBRON31”), high cholesterol (MEPS variable: “CHOLDX”), cancer (MEPS variable: “CANCERDX”), diabetes (MEPS variable: “DIABDX_M18”), joint pain (MEPS variable: “JTPAIN31_M18”), arthritis (MEPS variable: “ARTHDX”), and asthma (MEPS variable: “ASTHDX”). It also included health status (excellent, very good, good, or fair or poor; MEPS variable: “RTHLTH31/42/53”), mental health status (excellent, very good, good, or fair or poor; MEPS variable: “MNHLTH31/42/53”), instrumental activity of daily living limitations (yes or no; MEPS variable: “IADLHP31”), activity of daily living limitations (yes or no; MEPS variable: “ADLHLP31”), pain interference (none, little, moderate, or quite a bit or extreme; MEPS variable: “ADPAIN42”), and BMI (obese [≥30 kg/m^2^], overweight [25-30 kg/m^2^], or normal or underweight [<25 kg/m^2^]; MEPS variable: “ADBMI42”) [17].

#### Personal Health Practices Variables

In this study, personal health practices variables included the actions of an individual that have a preventative or consequential impact on health status, such as frequent moderate- to vigorous-intensity exercise (yes or no; MEPS variable: “PHYEXE53”), smoking status (smoker or nonsmoker; MEPS variable: “OFTSMK53”), whether they had ever received the COVID-19 vaccine (yes or no; MEPS variable: “COVAXEVR53”), whether they had received the influenza vaccine in the previous 12 months (yes or no; MEPS variable: “ADFLST42”), whether they had ever received the pneumonia vaccine (yes or no; MEPS variable: “ADPNEU42”), and whether they had ever received the shingles vaccine (yes or no; MEPS variable: “ADSHNG42”) [17].

#### External Environmental Variables

In this study, the only external environmental variable was region (northwest, midwest, south, or west; MEPS variable: “REGION22”) [17].

### Statistical Analysis

SAS (SAS Institute Inc) was used to perform the analysis of the data. As the MEPS is structured as complex survey data, the SAS survey procedures (eg, SAS PROC SURVEYFREQ and PROC SURVEYLOGISTIC), as well as cluster and stratum variables, were used. The cluster variable identifies the primary sampling unit, whereas the stratum variable identifies the stratification used in sampling. Furthermore, the appropriate weighting variable was used to obtain weighted population-based estimates [18]. The weighting variable provides the final annual person-level weight and helps calculate accurate SEs. Data that were coded as missing, inapplicable, refused, not ascertained, unknown, or incalculable (as indicated by negative codes in the codebook) were not included in this analysis. Differences between the characteristics of individuals in the 2 comparison groups (ie, those with MEPS-defined long COVID vs those without) were assessed using chi-square tests. A multivariable binomial logistic regression model was then developed to assess the association between each variable and having MEPS-defined long COVID. The event group was having MEPS-defined long COVID, and the reference group was not having MEPS-defined long COVID. An α value of .05 was selected a priori. Multicollinearity among variables was assessed and not detected.

### Ethical Considerations

The University of Arizona Institutional Review Board approved this study (00006677; July 16, 2025).

## Results

**Figure 2:**
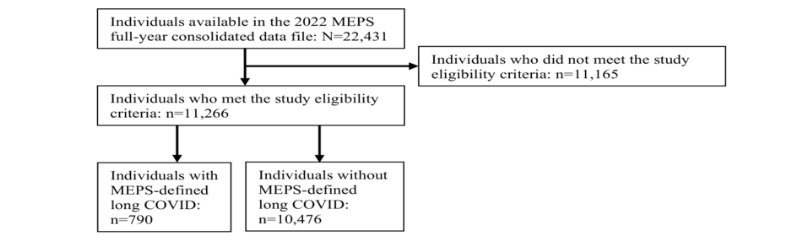
Study inclusion flowchart. MEPS: Medical Expenditure Panel Survey.

A total of 11,266 individuals were eligible for inclusion in this study. This represented a weighted population of 256,500,584 American adults. Of these 11,266 individuals, 790 (7%; weighted population=18,397,214) had MEPS-defined long COVID, whereas 10,476 (93%; weighted population=238,103,371) did not (Figure 2). The summary of complete and missing data for each variable in the study sample is reported in Table 1. Data were complete or largely complete for most variables except for BMI; pain interference; and having received the influenza, pneumonia, and shingles vaccines.

Regarding the predisposing variables, most American adults in this study were aged 18 to 64 years, female, White individuals, and not Hispanic. Regarding the enabling variables, most were married, had an educational level of more than high school, were employed, and had private insurance. A plurality had a high income. Regarding the need variables, the most common number of chronic conditions was 0, followed by 1 condition. Health status and mental health status were most often reported as very good or good. Most had no instrumental activity of daily living limitations, activity of daily living limitations, or pain interference. There were similar proportions of people in the obese, overweight, and normal or underweight BMI categories. Regarding the personal health practices variables, most reported doing frequent moderate- to vigorous-intensity exercise, being nonsmokers, receiving at least one COVID-19 vaccine, not receiving the influenza vaccine in the previous 12 months, never receiving the pneumonia vaccine, and never receiving the shingles vaccine. Regarding the external environmental variables, the most common region was the south. There were statistically significant differences between the MEPS-defined long COVID and no long COVID groups for most variables except for ethnicity, employment status, insurance type, exercise, smoking status, having received the influenza vaccine, having received the pneumonia vaccine, and region (Multimedia Appendix 1).

Among the predisposing variables, age of 65 years or above vs 18 to 64 years, male vs female sex, and Asian vs other or multiple races were associated with lower odds of having MEPS-defined long COVID. Among the enabling variables, being married vs never married and employed vs unemployed were associated with higher odds of having MEPS-defined long COVID. Among the need variables, having 0 vs 5 or more chronic conditions, 1 vs 5 or more chronic conditions, or 2 vs 5 or more chronic conditions was associated with lower odds of having MEPS-defined long COVID. Having excellent vs fair or poor health status or very good vs fair or poor health status was also associated with lower odds of having MEPS-defined long COVID. Having instrumental activity of daily living limitations was associated with higher odds of having MEPS-defined long COVID. Among the personal health practices variables, ever having received the COVID-19 vaccine was associated with lower odds of having MEPS-defined long COVID, whereas among the external environmental variables, residing in the south vs west region was associated with lower odds of having MEPS-defined long COVID. The Wald statistic for the final model was below 0.01. The concordance statistic for the final model was 0.70, indicating good predictive ability (Table 2).

**Table 1:** Summary of complete and missing data for each variable in the analytical sample.

Variable	MEPS^a^-defined long COVID, n	No MEPS-defined long COVID, n	Total, n
Age	790	10,476	11,266
Sex	790	10,476	11,266
Race	790	10,476	11,266
Ethnicity	790	10,476	11,266
Marital status	790	10,476	11,266
Educational level	790	10,476	11,266
Employment status	789 (missing=1)	10,469 (missing=7)	11,258 (missing=8)
Income	790	10,476	11,266
Insurance type	790	10,476	11,266
Number of chronic conditions	790	10,476	11,266
Health status	790	10,476	11,266
Mental health status	790	10,476	11,266
Instrumental activity of daily living limitations	790	10,440 (missing=36)	11,230 (missing=36)
Activity of daily living limitations	790	10,454 (missing=22)	11,244 (missing=22)
Pain interference	763 (missing=27)	10,033 (missing 443)	10,796 (missing=470)
BMI	756 (missing=34)	9813 (missing=663)	10,569 (missing=697)
Frequent moderate- to vigorous-intensity exercise	786 (missing=4)	10,400 (missing=76)	11,186 (missing=80)
Smoking status	788 (missing=2)	10,425 (missing=51)	11,213 (missing=53)
Ever received the COVID-19 vaccine	787 (missing=3)	10,400 (missing=76)	11,187 (missing=79)
Received the influenza vaccine in the previous 12 mo	771 (missing=19)	10,251 (missing=225)	11,022 (missing=244)
Ever received the pneumonia vaccine	469 (missing=321)	6161 (missing=4315)	6630 (missing=4636)
Ever received the shingles vaccine	470 (missing=320)	6180 (missing=4296)	6650 (missing=4616)
Region	790	10,476	11,266

^a^MEPS: Medical Expenditure Panel Survey.

**Table 2:** Variables associated with Medical Expenditure Panel Survey–defined long COVID (vs no long COVID) among American adults in this study^a^.

Variable			Odds ratio (95% CI)
**Predisposing variables**			
	Age: ≥65 vs 18-64 y	0.51 (0.35-0.75)	
	Sex: male vs female	0.64 (0.50-0.83)	
	Race: White vs other or multiple races	0.56 (0.25-1.25)	
	Race: Black vs other or multiple races	0.56 (0.24-1.30)	
	Race: Asian vs other or multiple races	0.25 (0.08-0.81)	
	Hispanic vs not Hispanic	1.20 (0.78-1.85)	
**Enabling variables**			
	Married vs never married	1.89 (1.10-3.24)	
	Widowed, divorced, or separated vs never married	1.64 (0.99-2.71)	
	Lower than high school education vs more than a high school education	0.95 (0.58-1.55)	
	High school education vs more than a high school education	1.25 (0.96-1.63)	
	Employed vs unemployed	1.54 (1.10-2.17)	
	Poor or low vs high income	1.06 (0.70-1.62)	
	Middle vs high income	0.96 (0.65-1.41)	
	Any private insurance vs uninsured	0.83 (0.36-1.94)	
	Public-only insurance vs uninsured	0.90 (0.38-2.10)	
**Need variables**			
	0 vs ≥5 chronic conditions	0.25 (0.11-0.59)	
	1 vs ≥5 chronic conditions	0.58 (0.35-0.98)	
	2 vs ≥5 chronic conditions	0.42 (0.26-0.66)	
	3 vs ≥5 chronic conditions	0.89 (0.58-1.37)	
	4 vs ≥5 chronic conditions	0.81 (0.52-1.25)	
	Excellent vs fair or poor health status	0.43 (0.23-0.81)	
	Very good vs fair or poor health status	0.53 (0.33-0.85)	
	Good vs fair or poor health status	0.75 (0.52-1.09)	
	Excellent vs fair or poor mental health status	0.74 (0.44-1.24)	
	Very good vs fair or poor mental health status	0.68 (0.42-1.10)	
	Good vs fair or poor mental health status	0.80 (0.51-1.26)	
	Instrumental activity of daily living limitations: yes vs no	2.53 (1.48-4.35)	
	Activity of daily living limitations: yes vs no	0.61 (0.30-1.26)	
	None vs quite a bit or extreme pain interference	0.98 (0.59-1.61)	
	Little vs quite a bit or extreme pain interference	1.16 (0.72-1.87)	
	Moderate vs quite a bit or extreme pain interference	1.09 (0.64-1.85)	
	Obese vs normal or underweight	1.29 (0.85-1.94)	
	Overweight vs normal or underweight	1.30 (0.89-1.90)	
**Personal health practices variables**			
	Frequent moderate- to vigorous-intensity exercise: yes vs no	1.10 (0.85-1.43)	
	Smoker vs nonsmoker	1.01 (0.70-1.47)	
	Ever received the COVID-19 vaccine: yes vs no	0.65 (0.43-0.97)	
	Received the influenza vaccine in the previous 12 mo: yes vs no	0.99 (0.74-1.34)	
	Ever received the pneumonia vaccine: yes vs no	1.19 (0.88-1.63)	
	Ever received the shingles vaccine: yes vs no	0.94 (0.71-1.23)	
**External environmental variables**			
	Northeast vs west region	0.73 (0.46-1.15)	
	Midwest vs west region	0.89 (0.60-1.30)	
	South vs west region	0.66 (0.45-0.96)	

^a^Wald statistic≤0.0001; *C* statistic=0.70.

## Discussion

### Principal Findings

The key findings from this study were the prevalence of MEPS-defined long COVID and the identification of variables statistically associated with higher or lower odds of having MEPS-defined long COVID among American adults. On the basis of the weighted analysis, approximately 18,397,214 American adults had MEPS-defined long COVID in 2022. This figure is consistent with data from the 2022 National Health Interview Survey, which found that 6.9% of American adults had ever had long COVID, although both figures are likely underestimates due to inconsistent definitions, lack of definitive diagnostic tests, and mild or indistinguishable symptoms of long COVID [19]. Furthermore, variables identified in this study associated with higher or lower odds of having MEPS-defined long COVID included 3 predisposing variables (age, sex, and Asian race), 2 enabling variables (marital status and employment status), 3 need variables (number of chronic conditions, health status, and instrumental activity of daily living limitations), 1 personal health practices variable (having ever received the COVID-19 vaccine), and 1 external environmental variable (residence in the south region). These findings are interpreted and discussed below.

In this study, among the predisposing variables, age of 65 years or above was associated with lower odds of having MEPS-defined long COVID. Other research on this is mixed. For example, in the meta-analysis by Tsampasian et al [8], older age was associated with an increased likelihood of developing long COVID, whereas in the studies by Ford et al [20] and Kim [12], prevalence of long COVID was lower in older adults than in other groups. Nevertheless, the data demonstrate that there are perhaps additional factors associated with long COVID in older adults that require further investigation. Speculatively, it may be the case that older adults were able to better isolate themselves during the COVID-19 pandemic and, therefore, were less likely to develop long COVID. It may also be the case that older adults do have long COVID but attribute the symptoms to the aging process.

Regarding the other predisposing variables, the finding that male sex was associated with lower odds of having MEPS-defined long COVID was very consistent with other research: female sex is consistently identified as a risk factor for long COVID [6-8]. Among the racial and ethnicity factors, Asian race vs other or multiple races was the only factor significantly associated with lower odds of MEPS-defined long COVID. These findings were somewhat unexpected, but again, the research is not very clear in this area. Some studies have shown that White individuals are more likely to have a long COVID diagnosis. A study by Shi et al [21] identified that White individuals were more likely than people from other racial groups to develop long COVID, and research by Pfaff et al [22] examining patterns in *International Classification of Diseases, 10th Revision, Clinical Modification*, coding identified that White, non-Hispanic individuals were more likely to receive a long COVID–related diagnosis than other groups. In contrast, Glassman [23] examined the US Census Bureau Household Pulse Survey data, finding that Hispanic and Black respondents were more likely than White respondents to report long COVID symptoms. Notably, in the analysis by Glassman [23], Asian respondents were the least likely to report long COVID symptoms, which does align with the findings of our study. Similarly, lower rates of long COVID were identified among Asian individuals living in the United States compared with other racial groups in a study by Wu et al [24], consistent with the findings of our study.

Regarding enabling variables, only being married and being employed were associated with higher odds of MEPS-defined long COVID relative to those who were never married or not employed. It was notable that educational level, income or poverty status, and health insurance type were not associated with MEPS-defined long COVID in this study. These findings are interesting given that other research, such as the studies by Subramanian et al [10] and Feldman et al [11], has shown higher rates of long COVID among individuals with higher levels of economic hardships vs those in more economically advantaged groups. Being married and employed are generally more stable economic positions. This suggests that additional factors may be at play, perhaps underreporting or underrecognition of some of the nonspecific symptoms of long COVID, such as fatigue, myalgias, or memory difficulties, in this population. Further research could provide more insights into this finding.

Results for the need variables are mostly consistent with data published elsewhere. Having fewer chronic health conditions was associated with lower odds of having MEPS-defined long COVID compared with having 5 or more conditions. Similarly, having an excellent or very good health status was associated with lower odds of MEPS-defined long COVID compared with having a fair or poor health status. Furthermore, having instrumental activity of daily living limitations was associated with higher odds of MEPS-defined long COVID compared with no limits. All of these factors suggest a positive association between worsening health status and likelihood of MEPS-defined long COVID, which does align with previously published data. One notable area of difference was BMI, which was not associated with MEPS-defined long COVID in this study, although it has been reported as an associated factor elsewhere [8,10].

Regarding personal health practices, only a previous receipt of the COVID-19 vaccine was associated with lower odds of MEPS-defined long COVID. These findings mirror what is published in the literature on reduction of risk of long COVID with receipt of the COVID-19 vaccine. In future studies, it would be interesting to explore data on the COVID-19 vaccine in greater detail beyond the dichotomous data available in the MEPS, for instance, to determine whether there is an association between the number of doses of the vaccine, accounting for the initial 2 doses and any additional doses administered over time, as well as factors such as waning immunity and the emergence of additional SARS-CoV-2 variants. In this study, smoking status, reported exercise habits, and receipt of the influenza or shingles vaccine were not statistically significantly associated with MEPS-defined long COVID.

In this study, residing in the south vs the west was associated with lower odds of MEPS-defined long COVID. No significant associations were identified between the other regions. This finding is unexpected given that a study by Blanchflower and Bryson [25] determined that Alabama, Mississippi, and West Virginia had the highest rates of long COVID among all US states. Additional factors such as population racial or ethnic makeup, health care access, practice patterns of health care providers, and perceptions of SARS-CoV-2 infection may also be affecting this finding. Given the large number of states included in each region, variations in state-level policies and practices related to COVID-19, and a myriad of other considerations, the region variable may not be the most informative to advance a meaningful understanding of long COVID.

Several limitations may influence the broad applicability of this study. As a cross-sectional study, causality cannot be determined, only association. These associations may represent predictive or consequential factors for long COVID. In addition, MEPS data are self-reported, and as such, are susceptible to recall and self-report bias. This study used MEPS-defined long COVID. Alternative definitions, such as patient-reported long COVID or a clinical diagnosis, may result in different findings. These data are from 2022, a point when long COVID had only recently been recognized by the medical community; it is possible that some participants in the MEPS were not familiar with symptoms of long COVID at that time and, therefore, may not have recognized the symptoms to report. Furthermore, this may have been especially pertinent for individuals who had less contact with the health care system in general (noninsured individuals or those with low income). Only variables captured by the MEPS were available for analysis, so other factors may provide additional insights into risks of long COVID. Relatedly, some data were only available in a binary form, which prevented a more nuanced examination of the variables. For instance, the binary nature of the COVID-19 vaccination variable may miss some of the variability in this factor given that there were initially 2 doses of the vaccine followed by additional doses as immunity waned over time and additional variants of the virus became apparent. Data were missing for some variables (due to responses being not reported, ineligible, or not ascertained), which may have biased the results and should also be considered when interpreting the findings of this study. The comparison group in this study was any American adult without long COVID regardless of whether the individual had previously had COVID-19. It is possible that some of the associations detected in this analysis reflect differences in COVID-19 exposure, testing, and diagnosis or access to health services. Future work may contemplate a similar study with a comparison group of individuals who had previously had COVID-19 but did not develop long COVID.

### Conclusions

This study analyzed self-reported MEPS data to identify the prevalence and factors associated with MEPS-defined long COVID among American adults. These findings offer additional insights into the limited literature available on long COVID in the United States. Some of the findings from this study reinforce what is already known about long COVID, whereas others indicate a contrary result that warrants further investigation of the complex environmental and social factors associated with MEPS-defined long COVID. Further research is necessary to monitor and address trends in the prevalence of this condition in the United States to further our understanding of the factors associated with it and to determine which factors cause long COVID and which are a consequence of it.

## Appendix

Multimedia Appendix 1 Characteristics of American adults with and without Medical Expenditure Panel Survey (MEPS)–defined long COVID included in the weighted analysis.

## Abbreviations

ABM: Andersen behavioral model
MEPS: Medical Expenditure Panel Survey

## Data Availability

The datasets generated or analyzed during this study are available from the corresponding author on reasonable request.

## References

[1] National Academies of Sciences, Engineering, and Medicine (2024). A Long COVID Definition: A Chronic, Systemic Disease State with Profound Consequences.

[2] Soriano JB, Murthy S, Marshall JC, Relan P, Diaz JV, WHO Clinical Case Definition Working Group on Post-COVID-19 Condition (2022). A clinical case definition of post-COVID-19 condition by a Delphi consensus. Lancet Infect Dis.

[3] Long COVID. Centers for Disease Control and Prevention.

[4] Luo S, Lai LY, Zhu R, Gao Y, Zhao Z (2025). Prevalence and duration of common symptoms in people with long COVID: a systematic review and meta-analysis. J Glob Health.

[5] Bartsch SM, Chin KL, Strych U, John DC, Shah TD, Bottazzi ME, O'Shea KJ, Robertson M, Weatherwax C, Heneghan J, Martinez MF, Ciciriello A, Kulkarni S, Velmurugan K, Dibbs A, Scannell SA, Shen Y, Nash D, Hotez PJ, Lee BY (2025). The current and future burden of long COVID in the United States. J Infect Dis.

[6] Oelsner EC, Sun Y, Balte PP, Allen NB, Andrews H, Carson A, Cole SA, Coresh J, Couper D, Cushman M, Daviglus M, Demmer RT, Elkind MS, Gallo LC, Gutierrez JD, Howard VJ, Isasi CR, Judd SE, Kanaya AM, Kandula NR, Kaplan RC, Kinney GL, Kucharska-Newton AM, Lackland DT, Lee JS, Make BJ, Min YI, Murabito JM, Norwood AF, Ortega VE, Pettee Gabriel K, Psaty BM, Regan EA, Sotres-Alvarez D, Schwartz D, Shikany JM, Thyagarajan B, Tracy RP, Umans JG, Vasan RS, Wenzel SE, Woodruff PG, Xanthakis V, Zhang Y, Post WS (2024). Epidemiologic features of recovery from SARS-CoV-2 infection. JAMA Netw Open.

[7] Hou Y, Gu T, Ni Z, Shi X, Ranney ML, Mukherjee B (2025). Global prevalence of long COVID, its subtypes, and risk factors: an updated systematic review and meta-analysis. Open Forum Infect Dis.

[8] Tsampasian V, Elghazaly H, Chattopadhyay R, Debski M, Naing TK, Garg P, Clark A, Ntatsaki E, Vassiliou VS (2023). Risk factors associated with post-COVID-19 condition: a systematic review and meta-analysis. JAMA Intern Med.

[9] Al-Aly Z, Davis H, McCorkell L, Soares L, Wulf-Hanson S, Iwasaki A, Topol EJ (2024). Long COVID science, research and policy. Nat Med.

[10] Subramanian A, Nirantharakumar K, Hughes S, Myles P, Williams T, Gokhale KM, Taverner T, Chandan JS, Brown K, Simms-Williams N, Shah AD, Singh M, Kidy F, Okoth K, Hotham R, Bashir N, Cockburn N, Lee SI, Turner GM, Gkoutos GV, Aiyegbusi OL, McMullan C, Denniston AK, Sapey E, Lord JM, Wraith DC, Leggett E, Iles C, Marshall T, Price MJ, Marwaha S, Davies EH, Jackson LJ, Matthews KL, Camaradou J, Calvert M, Haroon S (2022). Symptoms and risk factors for long COVID in non-hospitalized adults. Nat Med.

[11] Feldman CH, Santacroce L, Bassett IV, Thaweethai T, Alicic R, Atchley-Challenner R, Chung A, Goldberg MP, Horowitz CR, Jacobson KB, Kelly JD, Knight S, Lutrick K, Mudumbi P, Parthasarathy S, Prendergast H, Quintana Y, Sharareh N, Shellito J, Sherif ZA, Taylor BD, Taylor E, Tsevat J, Wiley Z, Williams NJ, Yee L, Aponte-Soto L, Baissary J, Berry J, Charney AW, Costantine MM, Duven AM, Erdmann N, Ernst KC, Feuerriegel EM, Flaherman VJ, Go M, Hawkins K, Jacoby V, John J, Kelly S, Kindred E, Laiyemo A, Levitan EB, Levy BD, Logue JK, Marathe JG, Martin JN, McComsey GA, Metz TD, Minor T, Montgomery AP, Mullington JM, Ofotokun I, Okumura MJ, Peluso MJ, Pogreba-Brown K, Raissy H, Rosas JM, Singh U, VanWagoner T, Clark CR, Karlson EW (2025). Social determinants of health and risk for long COVID in the U.S. RECOVER-Adult cohort. Ann Intern Med.

[12] Kim D (2025). A nationwide study of risk factors for long COVID and its economic and mental health consequences in the United States. Commun Med (Lond).

[13] Kananian S, Nemani A, Stangier U (2024). Risk and protective factors for the severity of long COVID - a network analytic perspective. J Psychiatr Res.

[14] Andersen RM (1995). Revisiting the behavioral model and access to medical care: does it matter?. J Health Soc Behav.

[15] Medical Expenditure Panel Survey (MEPS) survey background. Agency for Healthcare Research and Quality.

[16] (2024). MEPS HC 243 2022 full year consolidated data file. Agency for Healthcare Research and Quality.

[17] (2024). MEPS HC 243 2022 full year consolidated data codebook. Agency for Healthcare Research and Quality.

[18] Chen X, Gorrell P An introduction to the SAS® survey analysis PROCs. Social & Scientific Systems.

[19] Adjaye-Gbewonyo D, Vahratian A, Perrine CG, Bertolli J (2023). Long COVID in adults: United States, 2022. Centers for Disease Control and Prevention.

[20] Ford ND, Slaughter D, Edwards D, Dalton A, Perrine C, Vahratian A, Saydah S (2023). Long COVID and significant activity limitation among adults, by age - United States, June 1-13, 2022, to June 7-19, 2023. MMWR Morb Mortal Wkly Rep.

[21] Shi J, Lu R, Tian Y, Wu F, Geng X, Zhai S, Jia X, Dang S, Wang W (2025). Prevalence of and factors associated with long COVID among US adults: a nationwide survey. BMC Public Health.

[22] Pfaff ER, Madlock-Brown C, Baratta JM, Bhatia A, Davis H, Girvin A, Hill E, Kelly E, Kostka K, Loomba J, McMurry JA, Wong R, Bennett TD, Moffitt R, Chute CG, Haendel M (2023). Coding long COVID: characterizing a new disease through an ICD-10 lens. BMC Med.

[23] Glassman B (2023). Household pulse survey shows 31.1% reported symptoms three months or longer after they had COVID-19. United States Census Bureau.

[24] Wu Y, Sawano M, Wu Y, Shah RM, Bishop P, Iwasaki A, Krumholz HM (2024). Factors associated with long COVID: insights from two nationwide surveys. Am J Med.

[25] Blanchflower DG, Bryson A (2023). Long COVID in the United States. PLoS One.

