# Supplementary material for: Prevalence and Associations of Medical Expenditure Panel Survey–Defined Long COVID Among Adults: Cross-Sectional Study
Source: JMIR Form Res. 2026 Jun 22;10:e92323. doi: 10.2196/92323 (PMC13338675; doi:10.2196/92323)
Supplement: Multimedia Appendix 1 [file formative_v10i1e92323_app1.docx]

**Multimedia Appendix.** Characteristics of American adults with and without Medical Expenditure Panel Survey (MEPS)–defined long COVID included in the weighted analysis^a^.

| Variable | | | MEPS-defined long COVID, weighted % (95% CI) | No MEPS-defined long COVID, weighted % (95% CI) | Total, weighted % (95% CI) | *P* value |
| --- | --- | --- | --- | --- | --- | --- |
| **Predisposing variables** | | | | | | |
|  | **Age (y)** | | | | | <.001 |
|  |  | ≥65 | 17.9 (14.8-21.0) | 23.3 (22.0-24.5) | 22.9 (21.7-24.1) |  |
|  |  | 18-64 | 82.1 (79.0-85.2) | 76.7 (75.5-78.0) | 77.1 (75.9-78.3) |  |
|  | **Sex** | | | | | <.001 |
|  |  | Male | 37.6 (33.3-41.8) | 49.7 (48.6-50.8) | 48.8 (47.8-49.9) |  |
|  |  | Female | 62.4 (58.2-66.7) | 50.3 (49.2-51.4) | 51.2 (50.1-52.2) |  |
|  | **Race** | | | | | <.001 |
|  |  | Asian | 2.9 (1.5-4.2) | 7.2 (5.9-8.5) | 6.9 (5.7-8.1) |  |
|  |  | Black | 9.9 (6.5-13.4) | 12.7 (11.2-14.3) | 12.5 (11.1-14.0) |  |
|  |  | White | 83.8 (79.8-87.8) | 76.4 (74.4-78.5) | 77.0 (74.9-79.0) |  |
|  |  | Other or multiple races | 3.4 (1.6-5.1) | 3.6 (3.0-4.1) | 3.6 (3.1-4.1) |  |
|  | **Ethnicity** | | | | | .84 |
|  |  | Hispanic | 17.9 (14.3-21.5) | 17.5 (14.9-20.1) | 17.5 (15.0-20.0) |  |
|  |  | Not Hispanic | 82.1 (78.5-85.7) | 82.5 (79.9-85.1) | 82.5 (80.0-85.0) |  |
| **Enabling variables** | | | | | | |
|  | **Marital status** | | | | | <.001 |
|  |  | Married | 54.6 (50.0-59.3) | 50.5 (49.0-52.0) | 50.8 (49.4-52.3) |  |
|  |  | Widowed, divorced, or separated | 22.7 (19.1-26.3) | 19.2 (18.1-20.4) | 19.5 (18.4-20.6) |  |
|  |  | Never married | 22.7 (18.0-27.4) | 30.2 (28.9-31.6) | 29.7 (28.4-31.0) |  |
|  | **Educational level** | | | | | .04 |
|  |  | Lower than high school | 10.8 (8.0-13.7) | 12.1 (10.9-13.3) | 12.0 (10.9-13.1) |  |
|  |  | High school | 32.4 (28.1-36.6) | 27.0 (25.5-28.5) | 27.4 (25.9-28.9) |  |
|  |  | More than high school | 56.8 (51.9-61.7) | 60.9 (59.0-62.8) | 60.6 (58.7-62.5) |  |
|  | **Employment status** | | | | | .79 |
|  |  | Employed | 66.2 (62.2-70.2) | 66.8 (65.3-68.3) | 66.7 (65.3-68.2) |  |
|  |  | Unemployed | 33.8 (29.8-37.8) | 33.2 (31.7-34.7) | 33.3 (31.8-34.7) |  |
|  | **Income** | | | | | .03 |
|  |  | Poor or low | 28.4 (23.9-32.8) | 25.0 (23.3-26.7) | 25.2 (23.6-26.9) |  |
|  |  | Middle | 32.9 (27.4-38.4) | 29.3 (27.7-31.0) | 26.6 (27.9-31.3) |  |
|  |  | High | 38.8 (33.9-43.6) | 45.6 (43.5-47.8) | 45.1 (43.0-47.2) |  |
|  | **Insurance type** | | | | | .91 |
|  |  | Any private | 66.7 (62.8-70.7) | 67.4 (65.7-69.1) | 67.4 (65.7-69.0) |  |
|  |  | Public only | 26.1 (22.3-30.0) | 25.3 (23.8-26.7) | 25.3 (23.9-26.7) |  |
|  |  | Uninsured | 7.1 (4.6-9.6) | 7.3 (6.2-8.5) | 7.3 (6.3-8.4) |  |
| **Need variables** | | | | | | |
|  | **Number of chronic conditions** | | | | | <.001 |
|  |  | 0 | 17.8 (13.7-21.9) | 33.6 (32.0-35.1) | 32.4 (31.0-33.9) |  |
|  |  | 1 | 25.0 (20.5-29.5) | 25.9 (24.8-27.1) | 25.9 (24.8-27.0) |  |
|  |  | 2 | 18.2 (14.3-22.2) | 15.4 (14.5-16.3) | 15.6 (14.7-16.5) |  |
|  |  | 3 | 16.5 (13.6-19.3) | 10.5 (9.8-11.2) | 10.9 (10.3-11.6) |  |
|  |  | 4 | 9.1 (6.7-11.6) | 7.0 (6.4-7.5) | 7.1 (6.6-7.7) |  |
|  |  | ≥5 | 13.3 (10.4-16.3) | 7.6 (7.0-8.3) | 8.1 (7.4-8.7) |  |
|  | **Health status** | | | | | <.001 |
|  |  | Excellent | 10.1 (6.9-13.3) | 22.6 (21.1-24.0) | 21.7 (20.3-23.1) |  |
|  |  | Very good | 29.3 (25.1-33.5) | 36.1 (34.5-37.6) | 35.6 (34.1-37.0) |  |
|  |  | Good | 36.9 (32.3-41.6) | 30.0 (28.7-31.3) | 30.5 (29.2-31.8) |  |
|  |  | Fair or poor | 23.6 (19.5-27.7) | 11.4 (10.5-12.3) | 12.3 (11.3-13.2) |  |
|  | **Mental health status** | | | | | <.001 |
|  |  | Excellent | 16.5 (12.8-20.3) | 26.7 (25.3-28.1) | 26.0 (24.6-27.4) |  |
|  |  | Very good | 27.8 (23.5-32.0) | 34.4 (33.0-35.8) | 33.9 (32.5-35.3) |  |
|  |  | Good | 37.0 (32.2-41.8) | 29.6 (28.2-31.0) | 30.1 (28.8-31.5) |  |
|  |  | Fair or poor | 18.7 (14.6-22.9) | 9.3 (8.5-10.1) | 10.0 (9.2-10.8) |  |
|  | **Instrumental activity of daily living limitations** | | | | | <.001 |
|  |  | Yes | 6.8 (4.7-8.9) | 2.9 (2.5-3.3) | 3.2 (2.8-3.6) |  |
|  |  | No | 93.2 (91.1-95.3) | 97.1 (96.7-97.5) | 96.8 (96.4-97.2) |  |
|  | **Activity of daily living limitations** | | | | | .01 |
|  |  | Yes | 3.1 (1.7-4.5) | 1.6 (1.3-1.9) | 1.7 (1.4-2.0) |  |
|  |  | No | 96.9 (95.5-98.3) | 98.4 (98.1-98.7) | 98.3 (98.0-98.6) |  |
|  | **Pain interference** | | | | | <.001 |
|  |  | None | 48.8 (43.5-54.0) | 64.4 (62.9-65.8) | 63.3 (61.8-64.7) |  |
|  |  | Little | 26.5 (22.6-30.4) | 22.0 (20.8-23.2) | 22.3 (21.2-23.4) |  |
|  |  | Moderate | 11.9 (9.1-14.6) | 7.0 (6.4-7.7) | 7.4 (6.7-8.0) |  |
|  |  | Quite a bit or extreme | 12.8 (9.8-15.9) | 6.6 (5.9-7.2) | 7.0 (6.4-7.7) |  |
|  | **BMI** | | | | | <.001 |
|  |  | Obese | 42.8 (38.2-47.4) | 33.1 (31.7-34.6) | 33.8 (32.4-35.2) |  |
|  |  | Overweight | 32.4 (28.4-36.4) | 32.8 (31.6-34.0) | 32.8 (31.6-33.9) |  |
|  |  | Normal or underweight | 24.8 (20.2-29.4) | 34.1 (32.4-35.7) | 33.4 (31.8-34.9) |  |
| **Personal health practices variables** | | | | | | |
|  | **Frequent moderate- to vigorous-intensity exercise** | | | | | .26 |
|  |  | Yes | 49.2 (44.2-54.2) | 52.2 (50.6-53.8) | 52.0 (50.4-53.5) |  |
|  |  | No | 50.8 (45.8-55.8) | 47.8 (46.2-49.4) | 48.0 (46.5-49.6) |  |
|  | **Smoking status** | | | | | .17 |
|  |  | Smoker | 13.1 (9.9-16.3) | 11.0 (10.0-12.0) | 11.2 (10.2-12.1) |  |
|  |  | Nonsmoker | 86.9 (83.7-90.1) | 89.0 (88.0-90.0) | 88.8 (87.9-89.8) |  |
|  | **Ever received the COVID-19 vaccine** | | | | | .01 |
|  |  | Yes | 79.5 (74.6-84.3) | 84.9 (83.6-86.1) | 84.5 (83.2-85.8) |  |
|  |  | No | 20.5 (15.7-25.4) | 15.1 (13.9-16.4) | 15.5 (14.2-16.8) |  |
|  | **Received the influenza vaccine in the previous 12 mo** | | | | | .55 |
|  |  | Yes | 47.6 (42.7-52.5) | 49.1 (47.4-50.7) | 49.0 (47.3-50.6) |  |
|  |  | No | 52.4 (47.5-57.3) | 50.9 (49.3-52.6) | 51.0 (49.4-52.7) |  |
|  | **Ever received the pneumonia vaccine** | | | | | .61 |
|  |  | Yes | 49.3 (43.9-54.6) | 47.9 (45.9-49.8) | 48.0 (46.1-49.9) |  |
|  |  | No | 50.7 (45.4-56.1) | 52.1 (50.2-54.1) | 52.0 (50.1-53.9) |  |
|  | **Ever received the shingles vaccine** | | | | | .04 |
|  |  | Yes | 36.9 (31.9-42.0) | 42.4 (40.6-44.3) | 42.0 (40.3-43.8) |  |
|  |  | No | 63.1 (58.0-68.1) | 57.6 (55.7-59.4) | 58.0 (56.2-59.7) |  |
| **External environmental variables** | | | | | | |
|  | **Region** | | | | | .21 |
|  |  | Northeast | 16.4 (12.2-20.7) | 17.3 (14.6-20.0) | 17.2 (14.6-20.0) |  |
|  |  | Midwest | 23.0 (18.4-27.7) | 20.3 (17.4-23.3) | 20.5 (17.6-23.4) |  |
|  |  | South | 34.8 (28.9-40.7) | 39.0 (35.4-42.6) | 38.7 (35.1-42.2) |  |
|  |  | West | 25.8 (20.8-30.7) | 23.4 (20.5-26.3) | 23.6 (20.6-26.5) |  |

^a^Differences between groups were assessed using chi-square tests.
